# An Epidemiological Analysis of Vocal Fold Atrophy at the Tokyo Voice Center

**DOI:** 10.1055/s-0045-1810095

**Published:** 2025-10-16

**Authors:** Tomohiro Hasegawa, Yusuke Watanabe

**Affiliations:** 1 Tokyo Voice Center, International University of Health and Welfare, Tokyo, Japan; 2 Department of Epidemiology and Social Medicine, International University of Health and Welfare Graduate School of Public Health, Tokyo, Japan; 3 School of Medicine, International University of Health and Welfare, Narita, Japan

**Keywords:** vocal fold atrophy, voice handicap index, maximum phonation time

## Abstract

**Introduction:**

Vocal fold atrophy (VFA) is the most common voice disorder, and its morbidity increases with age.

**Objective:**

To update the epidemiological understanding of VFA by assessing prevalence and phonographic characteristics by age, sex, and occupation.

**Methods:**

A single-center retrospective chart review examined records of patients newly diagnosed with VFA from January 2020 to December 2022, analyzing age, sex, maximum phonation time (MPT), Voice Handicap Index (VHI), occupation, and voice disease complications.

**Results:**

Six hundred-and-ten patients (319 women, 291 men), ages 17 to 96 (median 64; mean 61.14), were included. Most were in their 70s, with a higher proportion of women overall, but a greater number of men in their 60s, and an equal sex distribution in their 70s. Among those ≥60, there were 185 women and 189 men. Some were diagnosed with atrophy at a young age. Most participants were unemployed, and functional dysphonia was the most common complication. A moderately negative correlation was found between VHI and MPT in unemployed men, while other analyses showed no or weak correlations between age, sex, occupation, and complications.

**Conclusion:**

VFA does not exclusively impact older adults. Early diagnosis and simple tests could potentially extend healthy life expectancy in unemployed men with VFA.

## Introduction

The global and national populations are ageing, leading to increased morbidity of vocal fold atrophy (VFA), a prevalent voice disorder among the elderly. Numerous studies have documented adverse health outcomes, 1 diagnostic method, 2 3 4 5 6 treatment options like voice therapy, 7 injection therapy, 8 and other treatments. 9 10 11 In addition, VFA can cause singing difficulties, 12 and Some studies have reported an association between cough and VFA, which can be problematic in many occupations that use the voice. 13 However, VFA remains less recognized compared to head and neck cancer, even among otolaryngologists. This lack of awareness possibly contributes to the limited research on VFA despite its rising prevalence due to ageing populations, resulting in insufficient evaluation and management practices. 14 Takano et al. 15 analyzed the relationship between age, maximum phonation time (MPT), and mean flow rate (MFR) in 72 VFA patients, but this study is over a decade old. Given the ageing demographic, the epidemiology of VFA likely has evolved. Furthermore, previous studies 14 15 had small sample sizes and did not assess the Voice Handicap Index (VHI), crucial for voice evaluation. Consequently, recent epidemiological data on VFA in our country is lacking. This study, therefore, extracted medical records, including age, MPT, VHI, occupation, and complications of newly diagnosed VFA patients at our institution, to explore their interrelations.

## Methods

Between January 2020 and December 2022, 610 patients (319 women and 291 men) visited our center. A voice specialist from the Department of Otolaryngology examined the vocal folds. The study lacked established diagnostic criteria for VFA, so the physician used laryngoscopy and stroboscopy to diagnose VFA based on the glottal gap in the closed phase and a comprehensive assessment of voice and other data. VFA was not diagnosed if the bilateral vocal processes did not contact during vocalization. We excluded patients previously diagnosed with VFA, those needing repeated consultations, and those treated with voice therapy (VT) or other interventions without initial voice examination (e.g., MPT, VHI), as these treatments could bias results. Additionally, we excluded patients who lacked initial visit voice data and those with VFA caused by vocal fold paralysis (e.g., bilateral or unilateral complete/incomplete paralysis). A complication was defined as a voice disorder in a patient diagnosed with VFA, and if two complications were present (e.g., vocal fold polyps and functional dysphonia), they were listed as duplicates.

Items extracted from the medical records included age, sex, MPT, VHI, occupation, and voice disease complications at the first diagnosis. Data was analyzed using R version 4.3.2. Correlations of MPT and VHI with the other variables were investigated. Correlations between parameters were categorized as follows: | r | = 0.7 to 1 indicated a strong correlation; | r | = 0.4–0.7, a moderate correlation; | r | = 0.2–0.4, a weak correlation; and | r | = 0 to 0.2, almost no correlation.

The study adhered to the Declaration of Helsinki principles and was structured as an opt-out study for participants. It received approval from our institution's Ethics Committee (BLINDED FOR REVIEW).

## Results

### Epidemiological Results

Table 1 summarizes the epidemiological findings. The study included 610 patients (319 women, 291 men), aged 17 to 96 years (median 64 years, mean 61.14 years), with the majority in their 70s. Approximately 25% were under 50, with some patients in their teens and 20s diagnosed with VFA. Women outnumbered men overall, but there were more men in their 60s and nearly equal numbers in their 70s. Among those aged ≥60 years, there were 185 women and 189 men, with a slightly higher number of men.

**Table 1:** Patient background characteristics

Age	No.	Female	Male	%
-19	2	1	1	0.3
20-29	29	20	9	4.8
30-39	49	24	25	8.0
40-49	67	39	28	11.0
50-59	89	50	39	14.6
60-69-	138	59	79	22.6
70-79-	171	87	84	28.0
80-89-	62	38	24	10.2
90-	3	1	2	0.5
	610	319	291	100
**Complications**	**No.**		**female**		**male**
None	431		229		202
Functional dysphonia	53		29		24
GERD*	29		17		12
Sulucs	22		5		17
Chorditis	19		10		9
Others	29		18		11
Duplication	27		11		16
	610		319		291
**Occupation**	**No.**	**Female**	**Male**	**%**
Unemployed	110	59	51	18.0
Company clerk	96	39	57	15.7
VP**	78	49	29	12.8
Homemakers	63	63	0	10.3
Management	58	12	46	9.5
Teacher	44	24	20	7.2
Desk-workers	26	6	20	4.3
Announcer	19	11	8	3.1
Medical profession	17	8	9	2.8
Service industry	17	7	10	2.8
Unidentified	15	7	8	2.5
Receptionist	15	10	5	2.5
Entertainment	11	3	8	1.8
Part-time job	6	5	1	1.0
Student	6	4	2	1.0
Shinto priest	5	2	3	0.8
Consultancy	4	0	4	0.7
Others	20	10	10	3.3
	610	319	291	100

* GERD: Gastro Esophageal Reflux Disease.

** VP: Vocal Performer.

Functional dysphonia was the most frequent complication, followed by gastroesophageal reflux disease (GERD), which, although a gastrointestinal disorder, was classified as a voice disorder complication due to its association with voice issues. Other complications included dysphagia, microvascular lesions, and voice tremors, with 27 cases involving multiple complications (e.g., functional dysphonia and GERD).

Most patients were unemployed, while the employed were primarily company clerks. The next largest group comprised professional vocal performers (VPs), including elite VPs (singers, actors, or other professional voice users), amateur singers, and music students. Our institution sees numerous voice users. Desk workers comprised lyricists, writers, painters, programmers, and real estate managers, categorized separately from company clerks due to unknown voice use levels. Fifteen patients did not disclose their occupations. Other participants included transportation workers, hairdressers, and cleaners.

### Relationship Between Age, VHI, and MPT

**Fig. 1:**
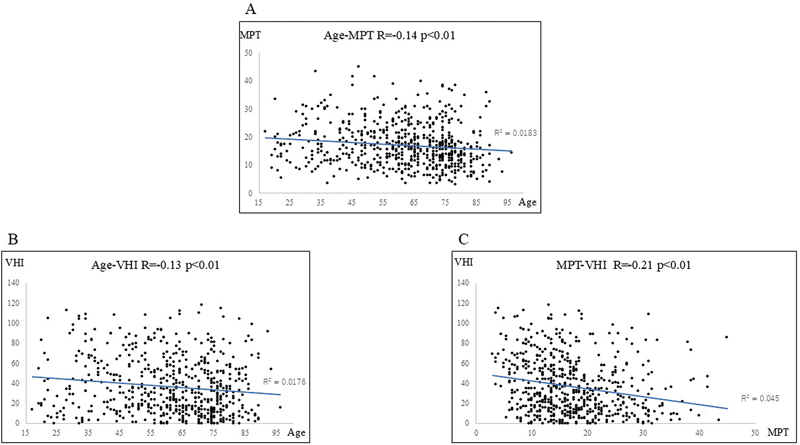
Relationship between ( **A** ): Age and MPT, ( **B** ): Age and VHI, and ( **C** ): MPT and VHI in all patients (total: 610; women: 319; men: 291). The correlation coefficients were R = −0.14, R = −0.13, and R = −0.21. MPT, maximum phonation time; VHI, Voice Handicap Index.

**Fig. 2:**
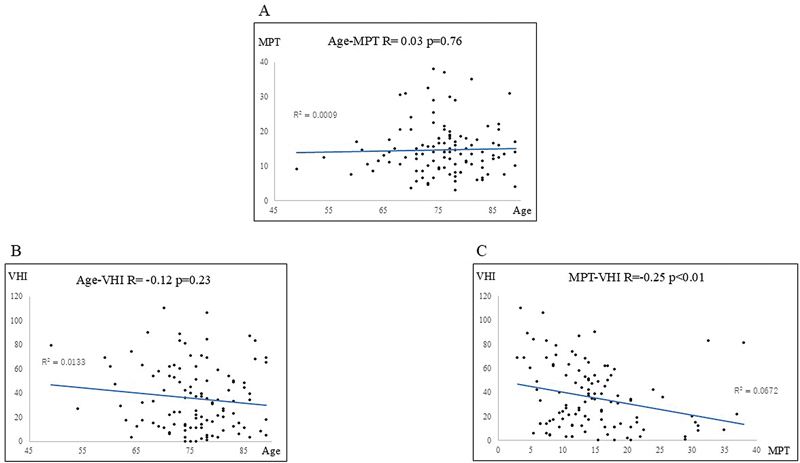
Relationship between ( **A** ): age and MPT, ( **B** ): age and VHI, and ( **C** ): MPT and VHI in unemployed patients (women: 59; men: 51). The correlation coefficients were R = 0.03, R = −0.12, and R = −0.26, respectively. MPT, maximum phonation time; VHI, Voice Handicap Index.

**Fig. 3:**
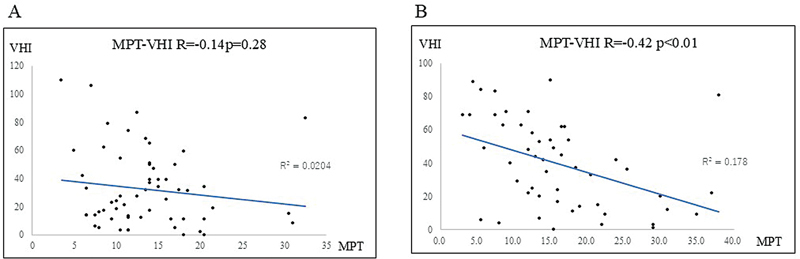
Relationship between MPT and VHI in unemployed women and B: men. The correlation coefficients were R = −0.14 for women and R = −0.42 for men. No correlation was found in women; however, a moderately negative correlation was observed in men. MPT, maximum phonation time; VHI, Voice Handicap Index.

**Fig. 4:**
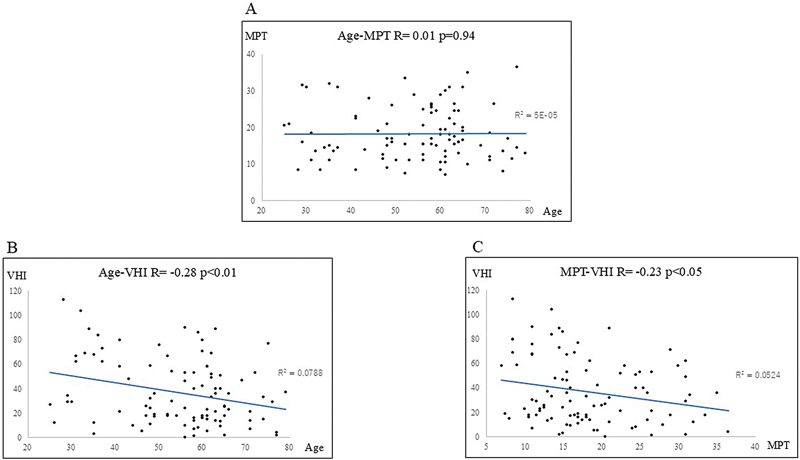
Relationship between ( **A** ): Age and MPT; ( **B** ): Age and VHI; ( **C** ): MPT and VHI in company clerks (total: 96; women: 39; men: 57). The correlation coefficients were R = 0.01, R = −0.28, and R = −0.23, respectively.

**Fig. 5:**
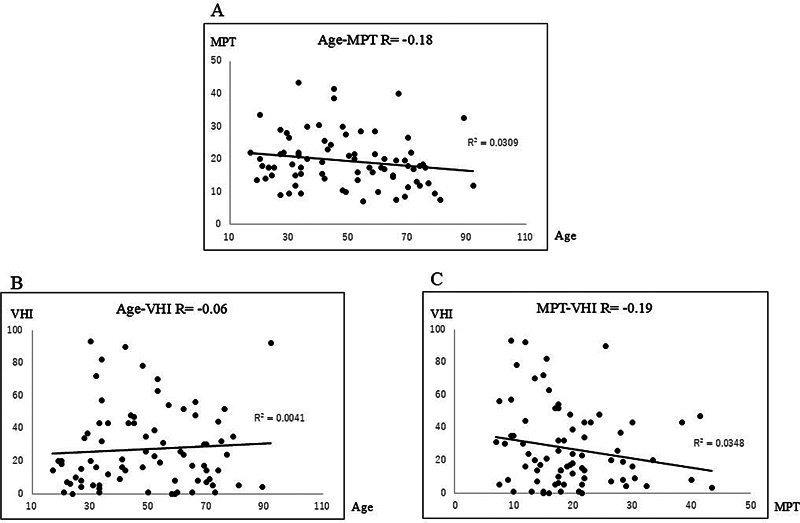
Relationship between ( **A** ): Age and MPT; ( **B** ): Age and VHI; ( **C** ): MPT and VHI in VP (total: 78; women: 49; men: 29). The correlation coefficients were R = −0.18, R = −0.06, and R = −0.19, respectively.

**Fig. 6:**
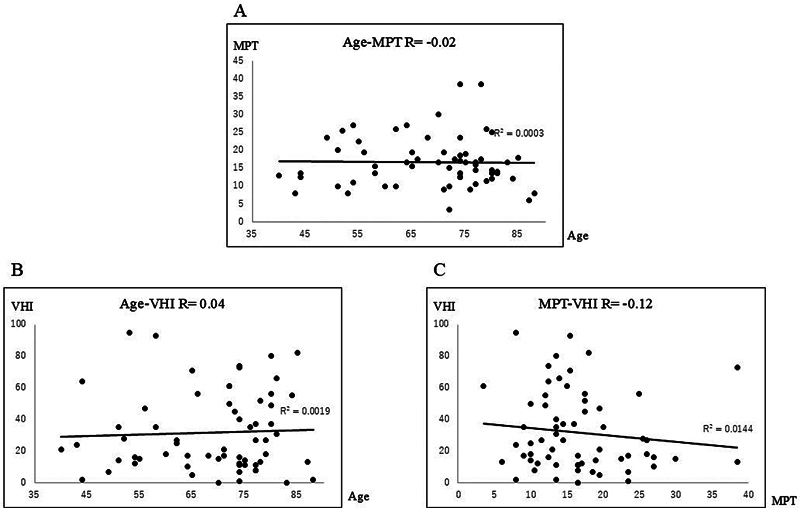
Relationship between ( **A** ): Age and MPT; ( **B** ): Age and VHI; ( **C** ): MPT and VHI in homemakers (total: 63; women: 63; men: 0). The correlation coefficients were R = −0.02, R = 0.04, and R = −0.12, respectively. MPT, maximum phonation time; VHI, Voice Handicap Index.

A negative correlation was observed between age and MPT ( Fig. 1A ; R = −0.14), age and VHI ( Fig. 1B ; R = −0.13), and MPT and VHI ( Fig. 1C ; R = −0.21) for all 610 patients. Figure 2 illustrates the relationship between age and MPT ( Fig. 2A ; R = 0.03), age and VHI ( Fig. 2B ; R = −0.12), and MPT and VHI ( Fig. 2C ; R = −0.26) in the 110 unemployed individuals. The relationship between MPT and VHI in unemployed women (R = −0.14) and men (R = −0.42) is depicted in Fig. 3 , with a moderate negative correlation found in men but not in women. For the 96 company clerks, the relationship between age and MPT ( Fig. 4A ; R = 0.01), age and VHI ( Fig. 4B ; R = −0.28), and MPT and VHI ( Fig. 4C ; R = −0.23) is shown in Fig. 4 . For 78 VPs, the relationship between age and MPT ( Fig. 5A ; R = −0.18), age and VHI ( Fig. 5B ; R = −0.06), and MPT and VHI ( Fig. 5C ; R = −0.19) is shown in Fig. 5 . For homemakers, the relationships between age and MPT ( Fig. 6A ; R = −0.02), age and VHI ( Fig. 6B ; R = 0.04), and MPT and VHI ( Fig. 6C ; R = −0.12) is shown in Fig. 6 . No correlation was found for complications.

## Discussion

### Epidemiological Results

Many patients in our study, both male and female, were in their 70s. About 25% were under 50, with some in their teens and 20s diagnosed with VFA. Previous studies 14 15 indicate VFA typically affects those in their 60s to 70s. Sugito et al. 14 noted that VFA begins in the 30s, a view we support. While VFA is more prevalent in older individuals due to ageing, some younger patients in our study were also diagnosed with VFA. Age did not significantly affect MPT and VHI, indicating that VFA can occur in younger individuals. Clinically, there are cases where vocal fold ageing does not align with actual age. The absence of a severity classification for VFA complicates therapeutic decisions. Misdiagnosing VFA in young patients can delay treatment and hinder voice improvement. Voice physicians should recognize VFA in young patients and consider less invasive treatments such as VT.

There were more women in this study, although VFA is less common in women than men. 14 15 Pontes et al. 16 found that age causes VFA in men but oedema in women. Consequently, women may be less likely to experience emphysematous hoarseness due to vocal fold oedema filling the glottal gap.

Most patients were unemployed, correlating with infrequent voice use. Employed patients predominantly worked as company clerks, whose tasks, such as document preparation, also required minimal voice use. Functional dysphonia was frequently observed. Koufman and Blalock 17 classified functional dysphonia into four types: type I (glottal gap during vocalization), type II (bilateral false vocal folds proximity due to compensatory vocalization), type III (moderate proximity between arytenoids and the petiole of the epiglottis), and type IV (very close proximity with barely observable vocal folds). All types were observed in this study.

### Relationship Between Age, MPT, and VHI by Occupation

Takano et al. 15 reported a negative correlation between age and MPT. We hypothesized that MPT would decline with age due to age-related changes in the vocal folds associated with VFA. However, our findings showed no significant correlation between age and MPT. Our institution, which specializes in voice issues, receives patients from various professions. As a result, the relationship between VHI, MPT, and age might be obscured by the inclusion of many VPs, who frequently use their voices. Therefore, we examined the relationships between age and VHI, age and MPT, and MPT and VHI through subgroup analyses by occupation and sex ( Table 2 ; Figs. 2 3 4 ). We identified a moderately negative relationship between MPT and VHI in unemployed men, while other groups showed no or weak correlations. This insight could be crucial for developing a severity classification of this disease. MPT, an objective measure, assesses the duration of the monovowel /a/ after maximal inhalation, reflecting one aspect of vocal performance but not the patient's satisfaction. In VFA, although MPT is significant, it should not be the sole indicator. It may be more effective to include MFR, bowing index, and other indices that reflect the glottal gap in the severity classification.

**Table 2:** Analysis results for Age, MPT, and VHI by occupation

All		610	319	291
		all	F	M
	MPT-Age	−0.14	−0.11	−0.19
	VHI-Age	−0.13	−0.14	−0.13
	MPT-VHI	−0.21	−0.21	−0.23
Unemployed		110	59	51
		all	F	M
	MPT-Age	0.03	0.01	−0.01
	VHI-Age	−0.12	−0.11	−0.1
	MPT-VHI	−0.26	−0.14	−0.42
Company clerk		96	39	57
		all	F	M
	MPT-Age	0.01	−0.08	0.04
	VHI-Age	−0.28	−0.38	−0.22
	MPT-VHI	−0.23	−0.28	−0.21
VP*		78	49	29
		all	F	M
	MPT-Age	−0.18	−0.03	−0.39
	VHI-Age	−0.06	0.05	0.10
	MPT-VHI	−0.19	−0.29	−0.05
Homemakers		63		
		all F		
	MPT-Age	−0.02		
	VHI-Age	0.04		
	MPT-VHI	−0.12		
Complications		431	229	202
		all	F	M
	MPT-Age	−0.13	−0.14	−0.16
	VHI-Age	−0.06	−0.07	−0.06
	MPT-VHI	−0.22	−0.18	−0.26
Over 50 years old		462	234	228
		all	F	M
	MPT-Age	−0.13	−0.12	−0.14
	VHI-Age	−0.09	−0.06	−0.12
	MPT-VHI	−0.23	−0.18	−0.29

* VP: Vocal Performer.

To examine the moderately negative relationship between MPT and VHI in unemployed men, we report the characteristics of retired men in our country. 94.4% of Japanese companies offer retirement at the age of 60-65. 18 Pre-retirement men often lack social interactions due to work commitments and struggle to engage socially post-retirement, and this social isolation is known to cause various disorders. 19 For example, social isolation causes hearing loss, 20 21 and depression, and suicide among the elderly are also major problems. 22 23 Additionally, the same sensory organ disorders, taste, 24 and smell disorders, 25 are also known to increase mortality, and it is thought that these disorders increase with decreased social interactions after retirement. We consider VFA as one of the causes of such social isolation. In addition, there are reports of VFAs increasing aspiration and pneumonia 26 27 Thus, reduced voice usage, especially among men, may, directly and indirectly, escalate mortality rates. In our super-ageing society, VFA in unemployed men warrants attention and should not be ignored.

The MPT is a straightforward test to measure how long an individual can sustain vocalization of monovowel sounds. While accurately assessing patient satisfaction is challenging, a moderately negative correlation exists between MPT and VHI in unemployed men. A low MPT in an unemployed male suggests dissatisfaction with his voice, warranting a referral to a specialist for potential VFA. Early interventions like VT 3 and intracordal injection 4 can enhance healthy life expectancy.

Conversely, no relationship was identified among age, MPT, and VHI in women, possibly due to higher social interaction and activity levels among women in our country, which may influence sex-related differences in VFA.

### Limitations

As a retrospective study based on medical record analysis, this research had certain limitations. First, the diagnostic criteria for VFA were not established during the study period, making it challenging to distinguish this condition from type 1 functional dysphonia or sulcus in young patients. Even with normal vocal folds, a glottal gap may appear during the closed phase of high-pitched tones, potentially increasing the diagnosis of VFA in women and young individuals. Additionally, VFA diagnoses were made by various voice disorder specialists in the Department of Otolaryngology, with individual physicians, varying in experience, diagnosing based on disease history, cause of onset, MFR, and other acoustic data, leading to potential variability in diagnostic criteria. Second, many patients at our institution are elite voice professionals (VPs) like singers and actors, prompting a subgroup analysis by occupation. Despite this, some selection bias may still exist. Given the numerous occupations today and no significant correlation between MPT and age, the analysis was stratified by occupation, but results may vary depending on occupation classification. Finally, although we described the characteristics of men who retired at 65, some unemployed men under 65 had not yet reached retirement age.

## Conclusion

We discovered that (1) VFA can affect young individuals, (2) VFA's prevalence may not vary by sex when young people are considered, and (3) MPT and VHI are correlated in unemployed men. We argue that VFA should not be viewed solely as a condition affecting men or older adults. With an ageing population, the incidence of VFA and aspiration pneumonia is anticipated to rise both nationally and globally. Utilizing simple tests like the MPT appropriately could potentially extend the healthy life expectancy of unemployed men diagnosed with VFA.
